# A Smartphone Intervention to Promote Time Restricted Eating Reduces Body Weight and Blood Pressure in Adults with Overweight and Obesity: A Pilot Study

**DOI:** 10.3390/nu13072148

**Published:** 2021-06-23

**Authors:** Malini Prasad, Keenan Fine, Allen Gee, Nandini Nair, Collin J. Popp, Bin Cheng, Emily N. C. Manoogian, Satchidananda Panda, Blandine Laferrère

**Affiliations:** 1 New York Nutrition Obesity Research Center, Division of Endocrinology, Columbia University Irving Medical Center, New York, NY 10032, USA; map2329@cumc.columbia.edu (M.P.); kf2617@cumc.columbia.edu (K.F.); ag4165@cumc.columbia.edu (A.G.); Nandini.Nair@nyulangone.org (N.N.); 2 Center for Healthful Behavioral Change, Department of Population Health, New York University Langone Health, New York, NY 10016, USA; Collin.Popp@nyulangone.org; 3 Department of Biostatistics, Mailman School of Public Health, Columbia University, New York, NY 10032, USA; bc2159@cumc.columbia.edu; 4 Salk Institute for Biological Studies, Regulatory Biology Department, La Jolla, CA 92037, USA; emanoogian@salk.edu (E.N.C.M.); panda@salk.edu (S.P.)

**Keywords:** meal timing, intermittent fasting, circadian rhythms, chrononutrition, time restricted eating

## Abstract

The goal of this study was to test the feasibility of time restricted eating (TRE) in adults with overweight and obesity. Participants (*n* = 50) logged all eating occasions (>0 kcal) for a 2-week run-in period using a smartphone application. Participants with eating duration ≥14 h enrolled in an open label, non-randomized, prospective 90-day TRE intervention, with a self-selected reduced eating window of 10 h. No dietary counseling was provided. Changes in anthropometrics, eating patterns and adherence after TRE were analyzed using *t*-tests or Wilcoxon Rank-Sum Test. The mean duration of the baseline eating window was 14 h 32 m ± 2 h 36 m (*n* = 50) with 56% of participants with duration ≥14 h. TRE participants (*n* = 16) successfully decreased their eating window from 16 h 04 m ± 1 h 24 m to 11 h 54 m ± 2 h 06 m (*p* < 0.001), and reduced the number of daily eating occasions by half (*p* < 0.001). Adherence to logging and to the reduced eating window was 64% ± 22% and 47% ± 19%, respectively. TRE resulted in decreases in body weight (−2.1 ± 3.0 kg, *p* = 0.017), waist circumference (−2.2 ± 4.6 cm, *p* = 0.002) and systolic blood pressure (−12 ± 11 mmHg, *p* = 0.002). This study demonstrates the feasibility and efficacy of TRE administered via a smartphone, in adults with overweight and obesity.

## 1. Introduction

Almost two thirds of American adults have overweight or obesity [1,2] and are at increased risk of developing associated chronic diseases, such as hypertension, type 2 diabetes [3] and cardiovascular disease [4,5,6,7]. Large-scale randomized-control trials have demonstrated the effectiveness of lifestyle changes and moderate (3–7%) weight loss in preventing the progression to type 2 diabetes and decreasing cardiovascular risk in people with prediabetes [8,9]. However, long-term sustainability of calorie restriction is difficult to achieve; therefore, alternate lifestyle strategies have been proposed [8,10,11,12,13,14,15] Smartphone applications (apps) [16] have shown efficacy as novel means of not only monitoring behavior, but also enforcing adherence to behavioral change, and can be used for weight loss interventions, such as time-restricted eating (TRE).

TRE, a form of intermittent fastingtable, is a novel lifestyle intervention that limits the duration of the daily eating window. Adults in the United States consume over 37% of calories after 6:00 p.m. and 50% of them have a daily eating window that exceeds 15 h [14]. Prolonged daily eating intervals in late hours, usually reserved for sleep, results in overeating and is associated with obesity [17,18,19]. Studies in rodents [20] and in humans [10,14,21,22,23,24] have shown that restricting the daily eating window leads to reduced adiposity, systemic inflammation and oxidative stress, improved insulin sensitivity, and longer sleep duration. The temporal aspect of food intake presents as an important modifiable behavior that may decrease predisposition to chronic diseases [25,26,27].

The primary aims of this pilot study were: (1) to test the feasibility of a TRE intervention, administered by a smartphone app, aim at reducing the eating window by 4 h in individuals with habitual prolonged eating window; (2) to determine the efficacy of a 90-day TRE intervention on reducing body weight and blood pressure, in adults with overweight and obesity. A secondary aim was to monitor the adherence to the intervention over time.

## 2. Materials and Methods

### 2.1. Participants

Participants were recruited from the Washington Heights neighborhood of northern Manhattan through community-level flyers and outreach, and through the websites RecruitMe and ResearchMatch. Inclusion criteria were: (1) men and women between the ages of 30–75 years; (2) BMI of 25–50 kg/m^2^, with or without known metabolic disorder; (3) having a smartphone; (4) residing in the New York City area. Exclusion criteria were: (1) shift workers; (2) planned travel across ≥2 time zones; (3) organ system dysfunction; (4) seizure disorder; (5) bariatric surgery within the past two years; (6) on weight loss medication; (7) severe psychiatric disorder. The Columbia University Institutional Review Board approved of the protocol. All participants provided written consent prior to enrollment. There was no financial compensation for participating in the study.

### 2.2. Study Design

This was an open label, non-randomized, prospective intervention with two phases: (1) a 2-week baseline observation run-in phase to identify individuals with eating window ≥14 h, followed by (2) a 90 day TRE intervention phase aiming to reduce the eating window to 10 h/d, while consuming their usual diet. The study was conducted over one year between June 2019 and June 2020.

After a phone screen, participants came for an in-person visit for consenting, medical history and physical examination. Participants also completed the Weight Efficacy Lifestyle (WEL) questionnaire to assess their degree of control and eating behavior [28] and the Ostberg Morningness-Eveningness questionnaire (MEQ) to assess their chronotype, i.e., whether one is a morning or evening person [29]. Height was measured to the nearest 1 cm, and body weight was measured in light clothing, after voiding, to the nearest 0.1 kg, with a digital scale with a stadiometer (SECA 769 Seca GmBH & Co. KG, Hamburg, Germany). Waist circumference (WC) was measured at the level of the umbilicus with a tape measure to the nearest 1 cm, in triplicate, by the same investigator; the average of the 3-measurements recorded. Systolic (SBP) and diastolic (DBP) blood pressure were measured manually with a manometer by the study physician 2 times, after the participant rested in sitting position for 5 min. The time of scheduled visit varied depending on *participants availability. The participants were then instructed to download the study app, myCircadianClock (mCC)* on their smartphone and received a 10 min tutorial where they learned how to use it to record their food intake. Participants were instructed to log all eating occasions (EO) in real time into the mCC app while following their usual diet. EOs included all foods and beverages excluding water.

Eligible participants with an eating window ≥14 h were offered to enroll in TRE, and attended a second in-person visit with repeated anthropometric measurements. Participants self-selected their 10-h eating window, starting within 3 h after usual wake time and ending at least 3 h before usual bedtime, and were instructed to consume all EO within this window. During the TRE intervention, participants received push notifications at fixed times one hour before the beginning and the end of the prescribed eating window. Random notifications were sent to remind participants to continue logging and the research coordinator, with access to the back end of the app, contacted the participants by push notification or text with reminders to use the app, if logging was absent (no logging for 2 days) or poor (1 login/day). The total number of random and added push notifications was not recorded. At the end of the 90-day intervention, participants returned for a final in-person visit with anthropometric measurements, repeat WEL and MEQ surveys and an “end-of-study” survey.

### 2.3. Questionnaires and Survey

The Weight Efficacy Lifestyle (WEL) questionnaire is based on 20 questions scored 0–9 utilizing a Likert scale, with a scale ranging from 0 to 180; the highest scores indicate a higher ability to control one’s eating and adopt weight management regimens [28,30]. The Horne and Ostberg Morningness-eveningness questionnaire (MEQ) is based on 19 questions. MEQ scores range from 16–86; higher scores indicated that an individual favored a morning chronotype [29]. We defined a score of 59–86 as ‘morning type’, 42–58 as ‘neither type’ and 16–41 as ‘evening type’. The post-study survey assessed satisfaction with the intervention, ease of using the app, and willingness to continue the TRE intervention.

### 2.4. myCircadianClock (mCC) App

MyCircadianClock is a validated [16] smartphone-based self-monitoring app. Participants record in real-time all food and beverages consumed throughout the day by taking photos of every food or beverage consumed using the app. After including an accompanying description, the photo automatically uploads into a cloud-based server. Once uploaded, the research team can remotely monitor and analyze the EO, eating duration, and meal content data. If the user misses or forgets to record a meal in real-time, they may log an EO later by entering a text description of the food or beverage and the associated time at which they consumed it.

### 2.5. Definition of Eating Window and Adherence

Eating patterns and adherence were derived from self-reported dietary intake data entered into the mCC app. Each participant’s eating window was calculated as the 95% interval of all EO entered into the mCC, as previously defined by Gill and Panda [16]. This is done to reduce day-to-day variation of one’s eating window. EOs were counted as distinct if logged >15 min apart from each other; logging events separated by ≤15 min were considered as a single EO. All food and beverages, excluding water, were taken into account when counting EO. Adherence to the usage of the app was assessed each day. A day was considered logging adherent if participants logged 2 events or more separated by at least 5 h in the app. The mean number of daily EOs was determined only on logging adherent days. During TRE, a day was considered window adherent if all EOs were consumed within the pre-defined 10 h eating window ±15 min on logging adherent days.

### 2.6. Statistical Analysis

Categorical variables were compared between groups with the chi-squared test. Continuous variables were first tested for normality by the Shapiro-Wilk test. Normally distributed data were represented as mean ± standard deviation for each group and comparison between groups via the two-sample *t*-test; the non-normally distributed data were reported as median ± inter-quantile range (IQR) and comparison between groups via the Wilcoxon two-sample test. Changes of outcome variables with the intervention were analyzed by the paired *t*-tests for normally distributed outcomes and by the signed rank tests for non-normal outcomes. Pearson correlations were used to determine the association between eating window duration and anthropometric changes with the intervention. Statistical analyses were performed in IBM SPSS Statistics (IBM SPSS Statistics for Windows, Version 27.0. IBM Corp, Armonk, NY, USA) and Prism (GraphPad Prism version 8.0.0 for Windows, GraphPad Software, San Diego, CA, USA). *p* < 0.05 was considered statistically significant.

## 3. Results

### 3.1. Baseline Run-in Period

**Figure 1:**
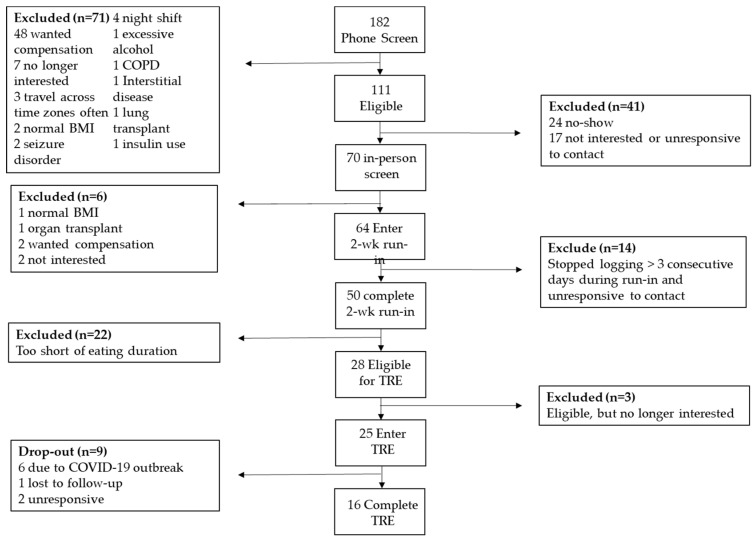
TREAT Pilot Study Consort Diagram.

**Figure 2:**
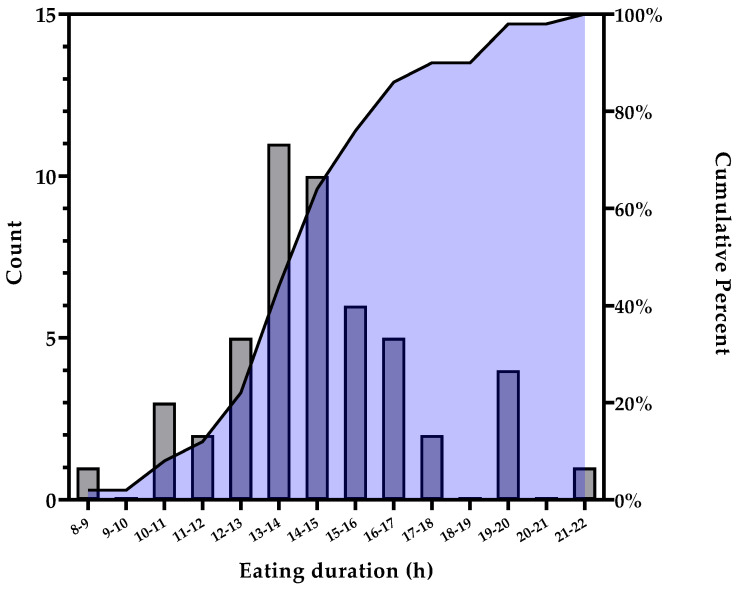
Eating duration during 2-week run-in period (*n* = 50). Frequency distribution (grey bars) and cumulative percentage (black line and purple shaded area) of eating duration.

Of the 64 enrolled participants, 50 (78.1%) completed the 2-week run-in phase (Figure 1). Non-completers either had poor logging, i.e., no logging for more than three consecutive days, declared they were not interested or did not respond to contact. Baseline participant characteristics are presented in Table 1. Participants were 51 ± 12 years of age, had a BMI of 31.0 ± 10.8 kg/m^2^, were predominantly women (82%), with 38% identifying as Hispanic or Latino. The distribution of the baseline eating windows is represented in Figure 2). The mean eating duration was 14 h 18 m ± 2 h 48 m with a first and final EO time of 9:09 ± 3:05 and 20:16 ± 2:34, respectively and an average number of EO/day of 5.3 ± 3.2 (Table 1). The logging adherence during the 2-week run-in period was 92.6% ± 28.6% of days (Table 1). More than half of participants (56%) had eating windows ≥14 h and were eligible for the TRE intervention. There were no significant age, gender and anthropometrics differences between individuals with eating duration ≥14 h and those with <14 h (Table 1). There were no differences between run-in completers and those who did not, except for an ethnic racial distribution (Table S1).

### 3.2. TRE Intervention

Of the eligible participants, 25 enrolled in TRE, 16 (64%) completed the 90-day intervention and were included in the analysis of eating and adherence patterns. Participants who dropped out during TRE intervention did so because of the COVID-19 pandemic outbreak in March of 2020 (Figure 1). There was no difference between TRE completers and non-completers, except for systolic blood pressure (*p* = 0.012) (Table S2). Due to COVID-19 lockdown, 2 participants who completed the 90-day intervention were unable to come in-person for final anthropometric measures.

Body weight decreased from 91.6 ± 17.1 kg to 90.1 ± 19.1 kg (*p* = 0.017, Table 2). Three participants (21.4%) lost ≥5% of body weight and 7 (50%) lost ≥3% of their body weight (Table S3). The median BMI decreased from 29.4 ± 8.2 to 28.9 ± 9.3 kg/m^2^ (*p* = 0.003) and waist circumference decreased from 98.9 ± 10.7 cm to 96.9 ± 7.5 cm (*p* = 0.002, Table 2). Systolic blood pressure decreased from 124.0 ± 27.5 mmHg to 114.0 ±17.3 mmHg (*p* = 0.002) (Table 2); 8/12 (66.7%) of the subjects reduced SBP by ≥10 mmHg (Figure S3).

**Figure 3:**
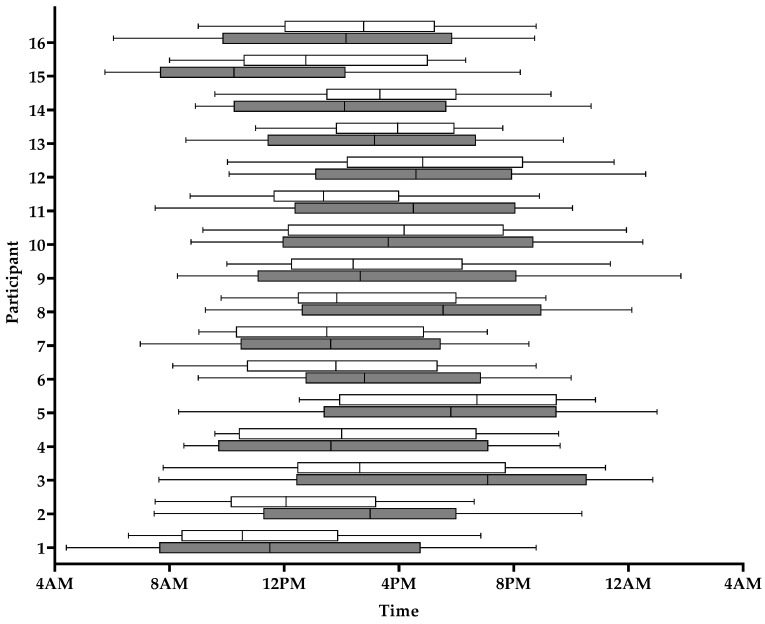
Individual 95% interval eating duration during baseline (grey bars) and during TRE (white bars) (*n* = 16).

On average, participants significantly reduced the duration of their eating window by 4 h 12 m from 16 h 06 m ± 1 h 24 m to 11 h 54 m ± 2 h 06 m (*p* < 0.001, Table 3, Figure 3). Most participants (*n* = 13, 81.3%) successfully reduced their eating window by ≥2 h, while 9/16 (56.3%) reduced their eating window by ≥4 h (Figure 3, Table S5) and 18.8% reduced their eating window to ≤10 h/d. The reduction of the eating window resulted from a delayed first EO from 8:56 ± 2:35 to 10:30 ± 2:23 (*p* < 0.001, Table 3) and an advanced final EO time from 20:12 ± 2:34 to 19:15 ± 2:35 (*p* < 0.001). The median time of all EO changed from 11:48 during baseline to 14:24 during TRE (*p* < 0.001). The overall number of daily EO decreased from 6.3 ± 1.9 during the run-in period to 3.9 ± 1.5 during the intervention (*p* < 0.001, Table 3). 

Overall, logging adherence decreased between baseline and TRE. During the 90-day TRE intervention, average logging and window adherence were 64% ± 22% and 47% ± 19%, respectively (Table 3). The majority (81.3%) of TRE completers were logging adherent for at least 50% of days; 25% adhered to logging on ≥80% of days (Table 1, Figure S1). Adherence to the reduced eating window was observed in 9/16 (56.8%) of the participants about 50% of the time during the entire intervention. One participant adhered to logging within the 10 h eating window ≥80% of the time (Table 1, Figure S1).

Both logging and window adherence decreased overtime during TRE (Table 1). The number of days with logging adherence decreased from 22.4 ± 5.1 in month one, to 19.4 ± 8.1 in month two and 14.3 ± 9.3 in the third and final month of the intervention (*p* = 0.016, Table S3). The number of days with window adherence also decreased overtime (*p* = 0.006) from 16.6 ± 5.9 (Month 1), to 13.4 ± 6.7 (Month 2) and 8.9 ± 6.8 (Month 3, Table S3). There was no correlation between the adherence measures and changes in anthropometrics measures (data not shown).

There was a non-significant increase in the WEL score from 130.3 ± 35.7 to 144.4 ± 33.0 (*p* = 0.132). Although non-significant, the MEQ score increased from 53.5 ± 14.8 to 57.5 ± 11.6 (*p* = 0.162), indicating a slight shift to a morning chronotype. Results from the end-of-study survey show that 55% found TRE easy to follow using the mCC app, and 82% stated they were likely or very likely to continue following TRE.

## 4. Discussion

The goal of this pilot study was to assess the feasibility of a TRE lifestyle intervention implemented via a smartphone app and to evaluate the efficacy of the intervention to reduce the eating window, body weight and blood pressure in adults with overweight and obesity. Our main findings are: (1) almost 60% of adults had habitual daily eating duration ≥14 h; (2) over half of the participants were able to reduce their eating window by >4 h; (3) A 10-h TRE intervention over 3 months resulted in modest but significant weight loss and clinically significant reduction in blood pressure; (4) overall, logging and window adherence was observed in roughly 65% and 50% of 90 days of the intervention, respectively.

Although long eating duration has been shown to be associated with obesity [17,18,19,31], we found no anthropometric differences between individuals eating ≥14 h and those with eating duration <14 h. Individuals with the longest eating window tended to have the lowest BMI (ns). These findings could be due to the small sample size and/or a bias of recruitment that targeted only participants with overweight or obesity, and may be confounded by differences in energy intake, which we did not measure.

We defined adherence to intervention in accordance with previous TRE studies [23]. Specifically, we examined patterns in logging adherence and window adherence. Overall, high logging adherence during the run-in period was not sustained throughout the intervention. These results are similar to previous behavioral weight loss studies showing decreased adherence over time [32,33]. The lack of monetary compensation may have contributed to the relatively low adherence. Both window adherence and logging adherence decreased throughout the study. Prior evidence from intermittent fasting interventions (TRE or alternate day fasting) [34] report substantially higher adherence rates (77% to 99%). This discrepancy could lie in the varying definitions of adherence and methods of capturing meal timing [14,35]. In our study, participants captured meal timing in real time with time-stamped photos of all eating events, with the adherence monitored daily via the back end by the coordinator. Other studies used self-report 7-day food diary [14], diary on timing of first and last meal during a 3 month intervention [35], or recall of self-reported adherence (yes/no) [36], without clear validation. While real-time, logs and recall methods to measure behavior and adherence are participants-dependent, and therefore have limitations, methods using real-time electronic time-stamped photos of meals may provide more accurate information on meal timing and adherence to the intervention. Diaries may introduce more bias [37] that may inflate adherence rates. Our low adherence rates could also be due to differences in participant behaviors and/or demographics.

In spite of the relatively low logging adherence, 57% of participants reduced their eating window by ≥4 h over the 90-day intervention. Our findings are in line with previous TRE studies that report a reduction in eating duration up to 5 h [23,24]. Participants reduced their eating duration by delaying their first EO by 1 h 24 m and advancing their final EO by 0 h 57 m. In our cohort, although all participants reduced their eating duration, only about 20% were able to reduce the eating duration to less than 10 h. In agreement to findings from Wilkinson et al. [23], while participants significantly reduced their eating duration, they had difficulties meeting the target restricted eating window. It may be very difficult for individuals with long eating durations to restrict their eating window to 6 h or 8 h for a prolonged intervention. This should be considered in designing future TRE interventions. Similar to other TRE studies using the mCC app [23,24], our participants decreased the number of daily EO from by nearly 40% during TRE. The reduced number of EO may result in reduced calorie intake and explain the weight loss.

TRE resulted in clinically and statistically significantly reduction of body weight, BMI, and waist circumference. This is in agreement with other human trials which demonstrated that restricting eating to 6 h, 8 h, or 10-h over 8–12 weeks is safe and effective in reducing body weight and fat mass [10,11,12,13,14,15,16,23], improving insulin sensitivity and β-cell function, and decreasing oxidative stress [12,38,39]. Our data is consistent with other studies that used the mCC app to test the efficacy of TRE lifestyle interventions [16,23,24]. In addition, we found a significant decrease in systolic blood pressure. Other studies have reported mixed findings on the effect of TRE on blood pressure [24,39,40]. This may be due to numerous confounders such as differences in participant characteristics and demographics between studies, adherence to the intervention, length of the prescribed eating window, and method of blood pressure measurement.

This pilot study had many strengths. We collected a large sample of eating data in an ambulatory setting (>4000 EOs) used to analyze eating patterns. We selected for the TRE intervention individuals with a long eating window based on remote monitoring of their eating patterns in free-living conditions. Eligible participants successfully reduced the duration of their eating window during the intervention. In spite of low adherence, TRE significantly reduced body weight and waist circumference with clinically significant decrease in systolic blood pressure. The burden of the intervention was relatively low.

This study had several limitations. It was not randomized and lacked a control group; however, each participant served as their own control. Because we did not quantify energy intake, changes in physical activity and/or diet composition during the TRE intervention, the improvements in cardiometabolic risk factors (blood pressure, body weight, waist circumference) cannot be attributed to either the circadian effects of TRE, increase in activity related energy expenditure or to a reduction in energy intake. In addition, we did not track the number of push notifications or its effect on adherence to the intervention. Attrition was also relatively high, as much of this study took place during the COVID-19 pandemic. Further research is needed to elucidate the mechanisms by which TRE improves blood pressure, independent of weight change, if this effect of TRE is confirmed in randomized trials.

## 5. Conclusions

We demonstrated the feasibility of implementing a 90 day TRE intervention in adults with overweight and obesity. The use of a validated smartphone app to deliver the intervention offers a low burden option. The observed effect on reduction of lower body weight, waist circumference, and blood pressure will need to be replicated in a randomized-control trial.

## Figures and Tables

**Table 1:** Run-in Baseline Participants Characteristics (*n* = 50).

Variable	All (*n* = 50)	Eating Duration ≤14 h (*n* = 22)	Eating Duration >14 h (*n* = 28)	*p*-Value
Age, y	51 ± 12	53 ± 13	50 ± 11	0.455
Gender, % (*n*)				
Male	18.0 (9)	22.7 (5)	14.3 (4)	0.441
Female	82.0 (41)	77.3 (17)	85.7 (24)	
Non-Hispanic, % (*n*)				
Non-Hispanic	62.0 (31)	63.6 (14)	60.7 (17)	0.833
Hispanic	38.0 (19)	36.4 (8)	39.3 (11)	
Race, % (*n*)				
White	56.0 (28)	63.6 (14)	50.0 (14)	0.266
Black	42.0 (21)	31.8 (7)	50.0 (14)	
Asian	2.0 (1)	4.6 (1)	(0)	
Other	0 (0)	(0)	(0)	
Height (cm)	165.3 ± 6.7	164.0 ± 6.0	166.4 ± 7.2	0.218
Weight (kg)	92.1 ± 18.2	93.5 ± 17.7	91.1 ± 18.8	0.645
^+^ BMI (kg/m^2^)	31.0 ± 10.8	33.1 ± 11.4	30.1 ± 9.2	0.287
^+^ SBP (mmHg) (*n* = 45)	115.0 ± 20.0	120.0 ± 30.0	113.5 ± 20.0	0.881
DBP (mmHg) (*n* = 45)	75.2 ± 9.6	75.3 ± 9.7	75.1 ± 9.7	0.950
^+^ WC (cm) (*n* = 49)	96.3 ± 15.7	95.3± 15.6	98.6 ± 16.6	0.968
Chronotype Score (*n* = 47)	55.0 ± 13.4	55.4 ± 11.7	54.8 ± 14.7	0.889
Chronotype, % (*n* = 47)				
Morning Type	40.4 (19)	35.0 (7)	44.4 (12)	0.673
Neither Type	42.6 (20)	50.0(10)	37.0 (10)	
Evening Type	17.0 (8)	15.0 (3)	18.5 (5)	
^+^ EO/Day	5.3 ± 3.2	3.6 ± 3.5	5.6 ± 2.1	**0.027**
^+^ Eating Duration	14 h 18 m ± 2 h 48 m	13 h 00 m± 2 h 00 m	15 h 30 m ± 2 h 36 m	**<0.001**
First EO Time (hh:mm)	9:09 ± 3:05	9:20 ± 2:15	8:54 ± 3:47	**0.003**
Final EO Time (hh:mm)	20:16 ± 2:34	19:53 ± 2:14	20:32 ± 2:44	**0.004**
^+^ Logging Adherence (%)	92.6 ± 28.6	85.2 ± 38.5	92.9 ± 21.4	0.134
WEL Score (*n* = 41)	125.3 ± 32.3	132.2 ±27.6	120.8 ± 34.8	0.269

BMI, body mass index; SBP, systolic blood pressure; DBP, diastolic blood pressure; WC, waist circumference; EO, eating occasion. Values are reported as mean ± SD or count (%) except ^+^ BMI, SBP, WC, EO/Day, Eating Duration, and Logging Adherence, which were not normally distributed by Shapiro-Wilk test and are reported as median ± IQR, *p*-value from Wilcoxon two-sample test. *p* < 0.05; significance in bold.

**Table 2:** Change in anthropometrics with TRE (*n* = 14).

Variable	Pre-TRE	Post-TRE	*p*-Value +	Median of the % Change
Weight (kg)	91.6 ± 17.1	90.1± 19.1	**0.017**	−2.2
BMI (kg/m^2^)	29.4 ± 7.5	28.9 ± 8.7	**0.017**	−2.3
WC (cm)	98.9 ± 10.7	96.9 ± 7.5	**0.002**	−2.1
* SBP (mmHg)	124.0 ± 27.5	114.0 ± 17.3	**0.002**	−10.1
* DBP (mmHg)	78.5 ± 9.3	75.0 ± 10.3	0.229	−10.0

BMI, body mass index; SBP, systolic blood pressure; DBP, diastolic blood pressure; Values are reported as median ± IQR since they were not normally distributed; *p* < 0.05; significance in bold. * *n* = 12. ^+^: from the signed rank test.

**Table 3:** Change in eating and adherence patterns between run-in and TRE (*n* = 16).

Variable	Run-in	Intervention	*p*-Value
Eating Duration	16 h 04 m ± 1 h 24 m	11 h 54 m ± 2 h 06 m	**<0.001**
First EO Time (hh:mm)	8:56 ± 2:35	10:30 ± 2:23	**<0.001**
Final EO Time (hh:mm)	20:14 ± 2:43	19:15 ± 2:35	**<0.001**
EO/Day	6.3 ± 1.9	3.9 ± 1.5	**<0.001**
Logging Adherence (%)	94 ± 10	64 ± 22	**<0.001**
Window Adherence (%)	N/A	47 ± 19	N/A

EO, eating occasions. Mean ± SD; *p* < 0.05; significance in bold.

## Data Availability

Data will be made available to investigators upon request to the corresponding author.

## Supplementary Materials

The following are available online at https://www.mdpi.com/article/10.3390/nu13072148/s1, Table S1: Baseline Characteristics of Run-in Completers vs. Non-completers, Table S2: Baseline Characteristics of TRE Completers vs. Non-completers, Table S3: Monthly Logging and Window Adherence during TRE, Figure S1: Individual Logging and Window Adherence during TRE, Table S4: Logging and Window Adherence by Month of TRE, Figure S3: Changes in Anthropometrics of Individual Completers of the TRE intervention, Table S5: Change in Anthropometrics during TRE.
