# Supplementary material for: A Smartphone Intervention to Promote Time Restricted Eating Reduces Body Weight and Blood Pressure in Adults with Overweight and Obesity: A Pilot Study
Source: Nutrients. 2021 Jun 23;13(7):2148. doi: 10.3390/nu13072148 (PMC8308240; doi:10.3390/nu13072148)
Supplement: Supplementary file 1 [file nutrients-13-02148-s001.zip › nutrients-1241661-supplementary.pdf]

Supplemental Figures Table of Contents

- **Supplemental Table S1.** Baseline Characteristics of Run-in Completers vs. Non-completers
- **Supplemental Table S2**. Baseline Characteristics of TRE Completers vs. Non-completers
- **Supplemental Table S3.** Monthly Logging and Window Adherence during TRE
- **Supplemental Figure S2.** Individual logging adherence (2a) and window adherence (2b) during TRE
- **Supplemental Table S4:** Logging and Window Adherence by Month of TRE
- **Supplemental Figure S3 (a-e):** Changes in Anthropometrics of Individual Completers of TRE Intervention
- **Supplemental Table S5:** Change in Anthropometrics during TRE

Supplemental Table S1. Baseline Characteristics of Run-in Completers vs. Non-completers

Variable		All (n=64)	Completers (n=50)	Non-completers (n=14)	p-value
Age, y		51 ± 12	51 ± 12	48 ± 9	0.342
Gender, % (n)					
	Male	22.7 (11)	18.0 (9)	14.3 (2)	0.745
	Female	82.8 (53)	82.0 (41)	85.7 (12)	
Non-Hispanic, % (n)					
	Non-Hispanic	62.5 (40)	62.0 (31)	64.3 (9)	0.876
	Hispanic	37.5 (24)	38.0 (19)	35.7 (5)	
Race, % (n)					
	White	56.0 (30)	56.0 (28)	14.3 (2)	**0.015**
	Black	51.6 (33)	42.0 (21)	85.7 (12)	
	Asian	1.6 (1)	2.0 (1)	0.0 (0)	
	Other	0.0 (0)	0.0 (0)	0.0 (0)	
Weight (kg)		93.1 ± 18.6	92.1 ± 18.2	96.7 ± 17.7	0.404
$^{+}$BMI (kg/m^2)		31.1 ± 11.8	31.0 ± 10.8	30.1 ± 9.2	0.655
$^{+}$SBP (mmHg) (n=57)		118.0 ± 20.0	115.0 ± 20.0	122.0 ± 24.0	0.609
DBP (mmHg) (n=57)		76.3 ± 10.2	75.3 ± 9.7	80.3 ± 11.8	0.120
$^{+}$WC (cm) (n=63)		98.9 ± 18.5	96.3 ± 15.7	101.2 ± 26.0	0.354

BMI, body mass index; SBP, systolic blood pressure; DBP, diastolic blood pressure; Values are reported as mean ± SD or count (%) except $^{+}$BMI, SBP, WC, which were not normally distributed by Shapiro-Wilk test and are reported as median ± IQR, p-value from Wilcoxon two-sample test.

$p<0.05$; significance in **bold**

Supplemental Table S2. Baseline Characteristics of TRE Completers vs. Non-completers

Variable		All (n=25)	Completers (n=16)	Non-completers (n=9)	p-value
Age, y		51 ± 10	51 ± 10	49 ± 11	0.708
Gender, % (n)					
	Male	16.0 (4)	25.0 (4)	0.0 (0)	0.102
	Female	84.0 (41)	75.0 (12)	100.0 (9)	
Non-Hispanic, % (n)					
	Non-Hispanic	60.0 (15)	56.3 (9)	66.7 (6)	0.610
	Hispanic	40.0 (10)	43.7 (7)	33.3 (3)	
Race, % (n)					
	White	36.0 (9)	37.5 (6)	33.3 (3)	0.707
	Black	60.0 (15)	56.3 (15)	66.7 (6)	
	Asian	4.0 (1)	4.6 (1)	0.0 (0)	
	Other	0.0 (0)	0.0 (0)	0.0 (0)	
Weight (kg)		91.6 ± 18.3	92.5 ± 18.3	90.1 ± 20.6	0.766
$^{+}$BMI (kg/m^2)		30.9 ± 10.4	30.3 ± 10.4	34.5 ± 14.6	0.419
$^{+}$SBP (mmHg) (n=19)		115.0 ± 20.0	124.0 ± 33.0	111.0 ± 40.0	**0.012**
DBP (mmHg) (n=19)		78.7 ± 8.7	79.0 ± 9.6	78.3 ± 7.6	0.868
WC (cm) (n=24)		100.0 ± 11.4	100.2± 9.0	99.8 ± 15.9	0.939

BMI, body mass index; SBP, systolic blood pressure; DBP, diastolic blood pressure; WC, waist circumference; Values are reported as mean ± SD or count (%) except $^{+}$Weight, BMI, SBP, DBP, WC, which were not normally distributed by Shapiro-Wilk test and are reported as median ± IQR, p-value from Wilcoxon two-sample test.

p<0.05; significance in **bold**

Values measured at screening visit

Supplemental Table S3. Monthly Logging and Window Adherence during TRE (n=16)

	Logging Adherence (Days/month)			Window Adherence (Days/Month)		
Participant	Month 1	Month 2	Month 3	Month 1	Month 2	Month 3
1	12	4	9	4	2	4
2	24	18	24	17	14	16
3	20	20	31	13	15	12
4	30	29	23	27	20	14
5	30	30	23	26	29	23
6	27	19	0	11	10	0
7	18	6	0	14	6	0
8	30	9	0	18	8	0
9	22	14	11	14	13	8
10	27	26	30	22	14	10
11	22	19	12	18	19	10
12	19	18	13	17	16	13
13	17	15	16	15	15	16
14	28	26	11	28	25	10
15	29	27	21	16	16	9
16	25	23	23	18	23	14

Note: Participants 6, 7, and 8 all had their final month occur during the holiday season

Supplemental Figure S2. Individual logging adherence (2a) and window adherence (2b) during the TRE intervention. Black bar: month 1; grey bar: month 2; white bar: month 3 (n=16).

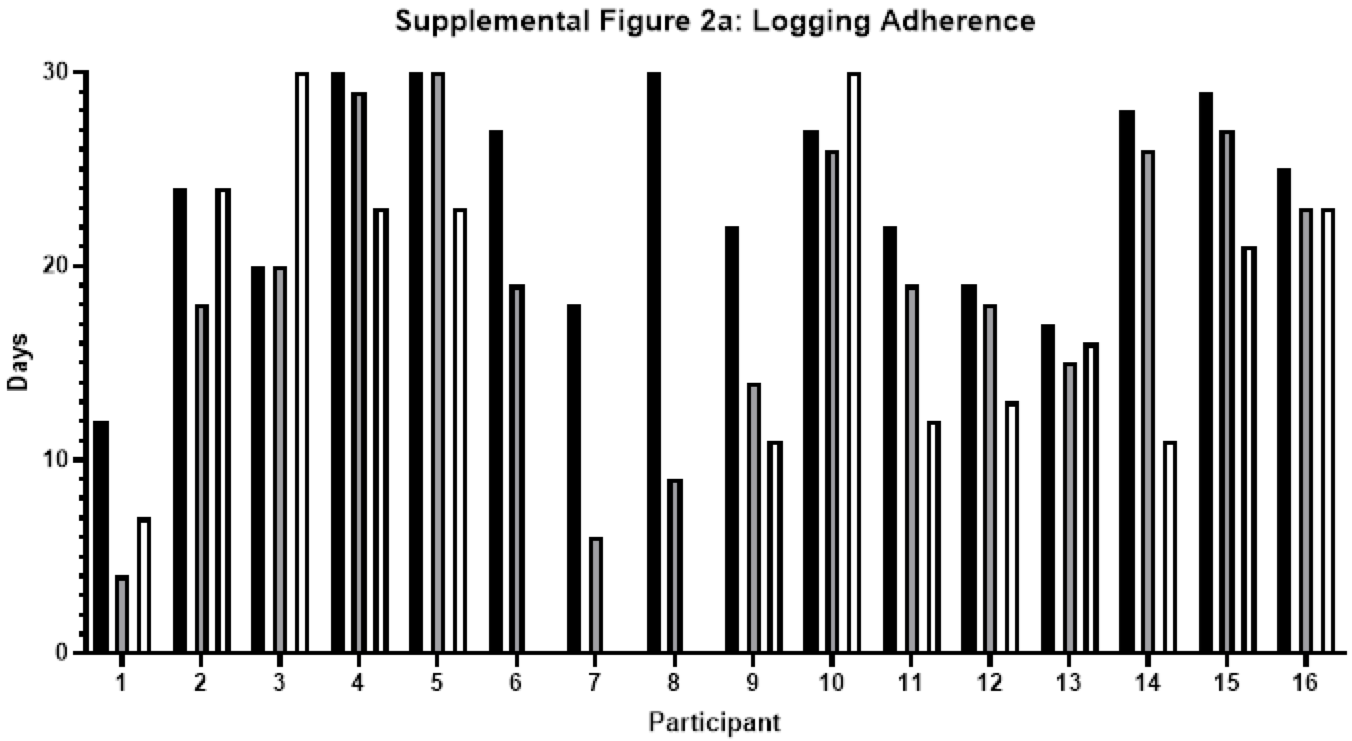

Supplemental Figure S2: Individual logging adherence (2a) and window adherence (2b) during the TRE intervention. Black bar: month 1; grey bar: month 2; white bar: month 3 (n=16).

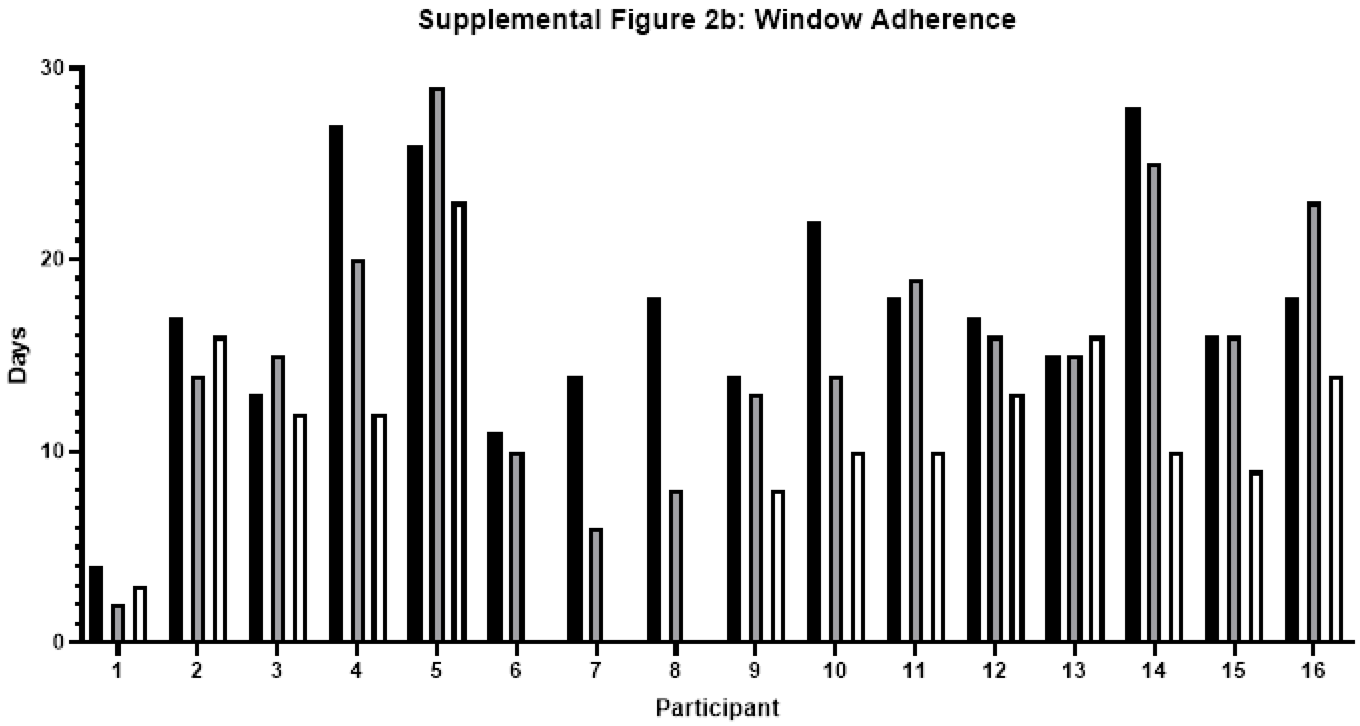

Supplemental Table S4: Logging and Window Adherence by Month of TRE (n=16)

Variable	Month 1	Month 2	Month 3	p-value
Logging Adherence (days)	22.4 ± 5.1	19.4 ± 8.1	14.3 ± 9.3	**0.016**
Window Adherence (days)	16.6 ± 5.9	13.4 ± 6.7	8.9 ± 6.8	**0.006**

Values are reported as mean ± SD

$p<0.05$; significance in **bold**

Supplemental Figure S3 (a-e): Changes in Anthropometrics of Individual Completers of TRE Intervention

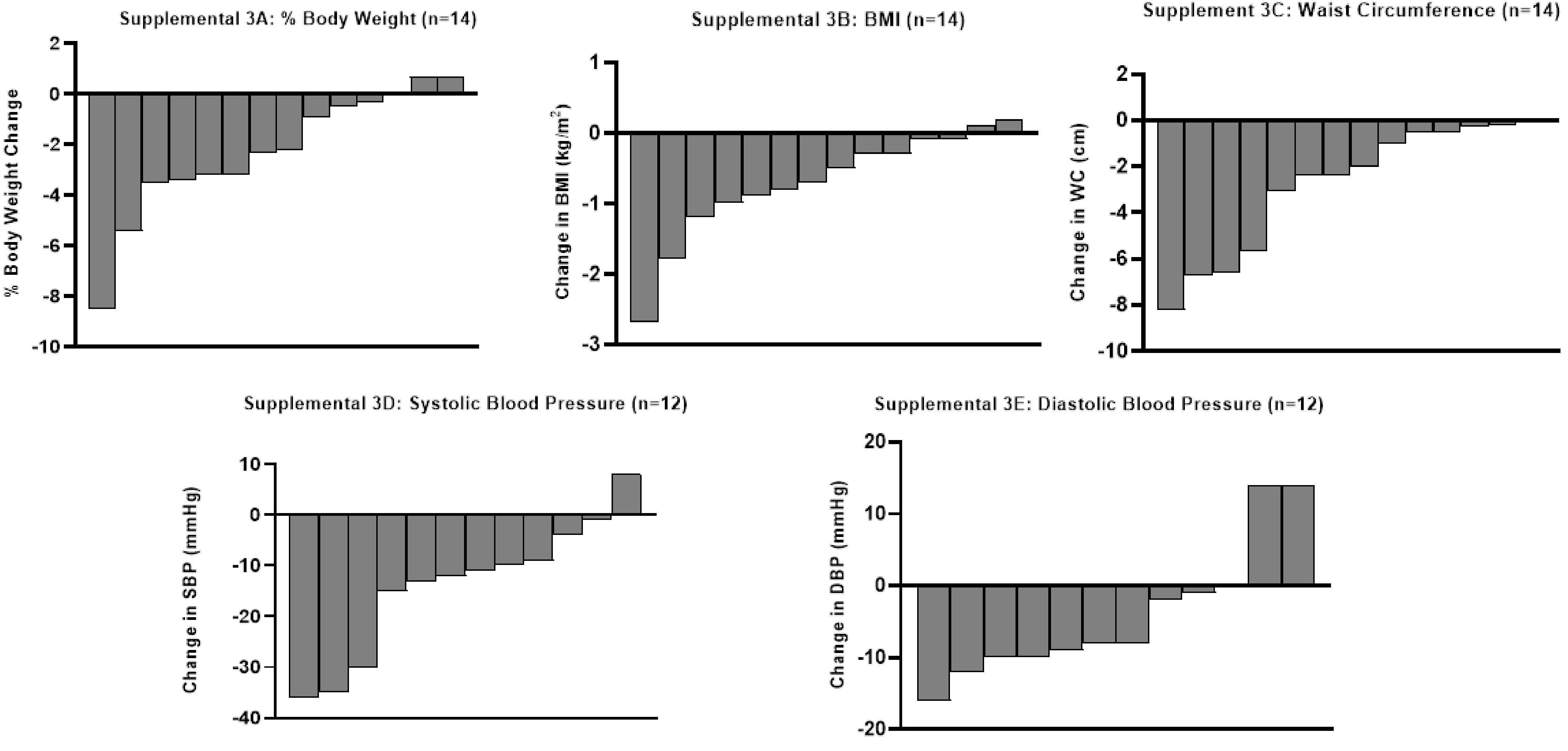

Supplemental Table S5: Change in Anthropometrics during TRE (n=16)

Participant	Reduction of Eating Duration (hh:mm)	% BW Change	BMI (kg/m^2)	WC (cm)	SBP (mmHg)	DBP (mmHg)
1	2h35m	**-3.2**	-1	**-5.7**	**-36**	-1
2	4h25m	**-5.4**	-1.8	**-6.6**	**-12**	**-8**
3	1h26m	**-3.4**	-0.7	-0.5	N/A	N/A
4	0h12m	**-8.5**	-2.7	**-6.7**	N/A	N/A
5	7h50m	**-3.5**	-0.8	**-3.1**	**-15**	**-10**
6	1h47m	-0.9	-0.1	0.1	**-13**	**-16**
7	4h55m	0.7	-0.3	-0.2	**-30**	**-8**
8	4h05m	-0.5	-0.3	-0.3	**-10**	**-10**
9	3h11m	0.1	0.1	-2	-4	0
10	6h36m	-2.2	-0.9	-2.4	-1	**-12**
11	2h45m	N/A	N/A	N/A	N/A	N/A
12	5h33m	-2.3	-1.2	**-8.2**	8	**14**
13	8h59m	-0.3	-0.1	-0.5	**-35**	**-9**
14	4h15m	**-3.2**	-0.5	-1	-9	-2
15	3h06m	N/A	N/A	N/A	N/A	N/A
16	4h13m	0.7	0.2	-2.4	**-11**	**14**
